# Maternal prenatal PTSD symptoms mediate the association between disrupted prenatal maternal representations of the child and infant social-emotional functioning

**DOI:** 10.3389/fgwh.2025.1606282

**Published:** 2026-01-09

**Authors:** Sarah M. Ahlfs-Dunn, Katherine L. Guyon-Harris, Diane Benoit, Alissa C. Huth-Bocks

**Affiliations:** 1 Department of Psychology, Eastern Michigan University, Ypsilant, MI, United States; 2 Department of Pediatrics, University of Pittsburgh School of Medicine, Pittsburgh, PA, United States; 3 Department of Psychiatry, University of Toronto, Toronto, ON, Canada; 4 Merrill Palmer Skillman Institute, Division of Research and Innovation, Wayne State University, Detroit, MI, United States

**Keywords:** disrupted, infant/toddler, maternal representations, mental health, prenatal, PTSD, social-emotional

## Abstract

**Introduction:**

There is limited research on disrupted maternal representations of the child, including possible mechanisms that may account for their impact on very young children's social-emotional well-being. Existing research suggests that maternal mental health symptoms, particularly those that may be related to experiences of interpersonal trauma, may be important to investigate.

**Method:**

Utilizing multi-method data, including the Working Model of the Child Interview, from a longitudinal study involving a community sample (*N* = 120) of women aged 18–42 who participated from their third trimester of pregnancy through 2 years post birth, the present study examined associations between disrupted prenatal maternal representations, infant and toddler social-emotional functioning, and perinatal (third trimester and 1-year post birth) maternal mental health symptoms (PTSD, depression, and anxiety).

**Results:**

Prenatal PTSD symptoms were the only maternal mental health symptoms significantly associated with degree of disrupted prenatal maternal representations and later infant and toddler social-emotional functioning. Mediation models revealed that maternal prenatal PTSD symptoms mediated the association between degree of disrupted prenatal maternal representations and infant, but not toddler, social-emotional functioning.

**Discussion:**

Findings highlight the importance of screening for maternal PTSD symptoms during the prenatal period as well as the value of early intervention when disrupted prenatal maternal representations are identified.

## Introduction

Both theory and research have identified the significant role that ‘maternal representations of the child’ have in impacting mothers’ cognitive, emotional, and behavioral functioning in relation to their children; this influence is notable as early as pregnancy. Maternal representations of the child (referred to simply as ‘maternal representations’ hereafter) are predominantly unconscious, interrelated thoughts and feelings about the self as a mother, the specific child of focus, and the relationship between mother and child (1–5). Maternal representations function to help the mother make sense of the child's behavior and respond accordingly, especially in attachment-relevant situations, i.e., those involving providing protection, care, and comfort (1, 6–8). As such, maternal representations are associated with child social-emotional outcomes during infancy and toddlerhood (9–12). Less well understood are the various mechanisms that account for or mediate the association between maternal representations and child social-emotional outcomes.

Maternal representations can be assessed prenatally and postnatally. The Working Model of the Child Interview [WMCI; (13)], a semi-structured interview, is one of the most common measures used to assess maternal representations. Researchers have identified four general categories of maternal representations that capture patterns in content, affect, organization, and consistency based on individuals’ narrative responses on a series of open-ended questions about their child and their relationship with that child. The first three representational categories are referred to as *balanced, disengaged,* and *distorted*. All three are considered ‘organized’ maternal representations of the child because a *consistent* set of mental processes is used to receive and interpret information and guide behavior in relation to providing protection and care for one's child (14), resulting in predictable caregiving responses that the child can anticipate and adapt to. Balanced is the most ideal type of maternal representation. It is least influenced by defensive processes; thereby allowing for revision and update as needed. It is characterized by, for example, flexibility, coherency, integration, sensitivity, joy, openness, and a balance between the positive and negative characteristics of the self as a mother, the child, and the relationship with the child. Disengaged and distorted maternal representations of the child are considered problematic and ‘non-balanced’ as they are characterized by defensive processes believed to interfere with the ability to provide responsive, sensitive care. These defensive processes result in deactivation of arousal and response to attachment cues from the child (disengaged), or heightened activation that is overwhelming or flooded (distorted) of the caregiving behavioral system. Disengaged maternal representations are characterized by, for example, coolness, distance, detachment, rigidity, devaluing intimacy and closeness in the relationship, emotional withholding, and indifference. In contrast, distorted maternal representations are characterized by incoherence, poor integration, heightened involvement and affect, and preoccupation with closeness (15–18).

Unlike the three organized types of maternal representations of the child, the fourth category of maternal representations, referred to as *disrupted*, is considered ‘disorganized’ and most problematic. There is *inconsistency* in mental strategies used to process information and guide behavior for providing protection and care of one's child, resulting in unpredictable and incoherent caregiving that a child cannot anticipate and adapt to. Disrupted maternal representations are characterized by, for example, contradictory response styles, failure to protect or provide comfort, role-confusion, intrusiveness, frightening behavior, fearfulness, helplessness, disorientation or dissociation, and/or extreme withdrawal (9). A coding scheme to use with the WMCI to assess disrupted maternal representations was developed much later than the coding scheme used to measure the original three organized classifications; thus, significantly less is known about disrupted maternal representations and their correlates. Given how problematic disrupted maternal representations may be for both parent and child outcomes, additional research is imperative. Research focusing specifically on *prenatal* disrupted maternal representations may, in fact, be most critical for early identification and treatment of parent-child relationship risks.

Maternal caregiving representations have been shown to be associated with a number of infant and toddler social-emotional outcomes. Specifically, prenatal and postnatal maternal representations are often found to be concordant with infant attachment classifications at 1 year of age, i.e., Balanced/Secure, Disengaged/Avoidant, Distorted/Ambivalent, Disrupted/Disorganized (9, 10, 13, 16). When infant attachment, defined as an infant's predominant strategy (or lack thereof) to maintain proximity with the caregiver, is measured along a continuum rather than categorically, disrupted prenatal and postnatal maternal representations have been found to be associated with less infant attachment security (19–21). Additionally, maternal representations categorized as disengaged or distorted, or containing non-balanced qualities (e.g., less joy, more anger, greater restriction, and less coherency), have been found to be associated with maladaptive or poor social-emotional child outcomes, such as mental health referrals and diagnoses (22–24), less positive affect/more negative affect (e.g., sober and withdrawn mood, anger), less attention seeking/contact maintenance when distressed, poorer quality of play, poorer attentional skills (11, 12, 25), and lower social-emotional competence (26). Degree of disrupted prenatal maternal representations, specifically, has been found to be associated with poorer infant and toddler social-emotional functioning based on parent reports (27).

Different mechanisms likely account for the impact of maternal representations on infant and toddler social-emotional functioning. For example, researchers have proposed that caregiver qualities, such as sensitivity (24), affective regulatory style (28), display of emotion (29), expression of attributions and projective identification (30) may, at least, partially account for infant and toddler social-emotional outcomes when balanced, disengaged, and distorted maternal representations are examined.

Considerably less is known about the mechanisms that help explain associations between disrupted maternal representations and infant and toddler social-emotional functioning. However, research that we have conducted on this representational category provides initial findings that require further examination. Specifically, we have found that severity of mothers’ childhood interpersonal trauma, i.e., events or experiences that threaten a sense of safety (psychological and/or physical) and that occur within close relationships, is associated with degree of disrupted prenatal maternal representations of the child (19). We have also found that the degree of atypical or disrupted maternal behavior (strange, frightening, or incoherent caregiving behaviors) at 1-year post birth partially mediates the association between degree of disrupted prenatal maternal representations and infant and toddler social-emotional functioning (27). More work is needed to identify other potential key mechanisms in order to guide assessment and interventions with families at high risk for relational and social-emotional impairments.

One potential mechanism that has yet to be explored is maternal mental health symptoms. History of childhood interpersonal trauma is not only associated with disrupted prenatal maternal representations, as noted above, but has been associated with an increased likelihood of maternal mental health symptoms in many studies [e.g., (31, 32)]. History of childhood interpersonal trauma is also associated with disrupted maternal behavior during the perinatal period (89), which, as also noted above, has associations with disrupted prenatal maternal representations and infant and toddler social emotional functioning. Thus, maternal mental health symptoms, particularly those that may be related to experiences of interpersonal trauma, such as posttraumatic stress disorder (PTSD), depression, and anxiety symptoms, may play an important role in the pathway between disrupted maternal representations and infant and toddler social-emotional functioning.

Although research has not yet investigated associations between disrupted maternal representations and maternal mental health symptoms, related research has shown that distorted maternal representations have been linked to a history of Major Depressive Disorder, higher levels of depressive symptoms, more severe PTSD symptoms as a result of interpersonal trauma, and symptoms of hostility (12, 33–37). Further, during the perinatal period, the prevalence rates of various mental health symptoms (e.g., PTSD, depression, anxiety) experienced by mothers are high (38–40), and perinatal maternal mental health symptoms are associated with poorer infant and toddler social-emotional functioning (41–44).

The primary aim of the present study was to better understand the role of maternal mental health in the relationship between disrupted maternal representations of the child and very young children's social-emotional well-being. Therefore, we examined associations between disrupted prenatal maternal representations of the child, infant and toddler social emotional functioning, and perinatal (third trimester and 1-year post birth) maternal mental health symptoms (i.e., PTSD, depression, anxiety). We then explored maternal mental health symptoms as a mediator between disrupted prenatal maternal representations of the child and infant and toddler social-emotional functioning. We hypothesized that prenatal and postnatal symptoms of PTSD, depression, and anxiety would each mediate the association between disrupted prenatal maternal representations and infant and toddler social-emotional functioning.

## Method

### Participants

As detailed in previous publications (19, 27), participants included 120 women who participated in a prospective, longitudinal study (data collected 2007–2011). Women entered the study during the third trimester of pregnancy and participation continued through 2 years post birth. At study entry, women were 26.2 years old on average (*sd* = 5.7). Forty-seven percent identified as African American, 36% as White, 12% as biracial, and 5% as other. The sample was predominantly economically disadvantaged with a median monthly income of $1,500 and high rates of social service utilization: 76% public health insurance, 73% Women, Infants and Children social service program (WIC), 52% food assistance, 17% public supplemental income. Seventy-eight percent of participants were currently in a relationship at study entry; the majority (63%) were never married, 28% were married, and 9% were divorced or separated. Twenty percent of participants attained an educational level of high school/GED or less, 44% completed some college courses or had a trade school degree, and 36% had a college or higher degree. Forty-eight percent were first time parents and 30% were experiencing their first pregnancy. The sample reported high rates of trauma exposure across their lifetimes with 90% experiencing at least one form of childhood maltreatment, 81% experiencing intimate partner violence (IPV) during pregnancy, and 90% experiencing lifetime IPV.

Recruitment occurred in urban and suburban communities in the Midwestern United States. Participants were recruited via fliers advertising a study about parenting placed at areas primarily serving low-income or high-risk pregnant populations including several community-based health clinics serving low-income and/or uninsured individuals (23%), WIC (18%), student areas in one regional-level university and one community college (16%), a “community baby shower” sponsored by local social service programs (11%), Head Start and local daycare programs (7%), subsidized and/or temporary housing facilities (7%), second-hand, donation centers for pregnant women and young children (5%), and a parenting class (2%). Eleven percent were recruited through word of mouth.

### Procedures

Data from three research visits were used in the present study: third trimester and 1- and 2-years post birth of the target child. The majority of research visits at each data collection time point were conducted in the participant's home (81%, 92%, and 92%, respectively), and the target child was present for the two post birth research visits. Informed and written consent was obtained at each data collection visit, and participants were compensated ($25 Target gift card after the third trimester research visit; $50 cash and a baby gift after the 1-year post birth research visit; and $40 cash, $10 Target gift card, and a baby gift after the 2-year post birth research visit). IRB approval was obtained for the larger study at the institution where the data were collected.

### Measures

#### Disrupted prenatal maternal representations of the child

Disrupted prenatal maternal representations of the child were assessed based on administration and coding of the Working Model of the Child Interview [WMCI; (13, 45)], a semi-structured interview of 37 open-ended questions about the child, the self as a mother, and the relationship with the child. The validity and reliability of the WMCI as a measure of maternal representations of the child has been well established (90).

The WMCI-Disrupted coding scheme (9) was used to identify prenatal disrupted maternal representations of the child. The WMCI-Disrupted coding scheme is based on the Atypical Maternal Behavior Instrument for Assessment and Classification [AMBIANCE; (46)]. Use of the WMCI-Disrupted coding scheme involves coding each transcript in its entirety, using 7-point Likert type scales, along five dimensions: (affective communication errors, role/boundary confusion, fearfulness/dissociation/disorientation, intrusiveness/negativity, and withdrawal) and then assigning an overall classification (disrupted or not-disrupted classification), an overall total score (degree of disruption ranging from 1 = *not disrupted* to 7 = *severely disrupted*), and a subtype classification reflecting the pattern of disruption (88). A more detailed description of the five dimensions and coding scheme can be found elsewhere (9). Previous research has demonstrated adequate convergent and discriminant validity, inter-rater reliability, and stability of the disrupted/not-disrupted classification over an 8-month period (9, 20, 21, 47).

In the present study, WMCIs administered during the third trimester of pregnancy were coded by the first author using the WMCI-Disrupted coding scheme. The third author and developer of the coding scheme (D. Benoit) double coded approximately 20% (*n* = 24) of the transcripts to establish inter-rater reliability. There was 96% agreement on the disrupted/not-disrupted classification (*κ* = .65). The intra-class correlation for the total/degree of disruption was .70 [*κ* = .69]. The total/degree of disruption score was used in the present study analyses.

#### Infant and toddler social-emotional functioning

Infant and toddler social-emotional functioning was assessed via the 42-item, parent-report, Brief Infant-Toddler Social and Emotional Assessment [BITSEA; (48)]. Items are rated on a 3-point scale regarding child behavior over the last month (0 = *not true/rarely*, 1 = *somewhat true/ sometimes*, 2 = *very true/often*). Thirty-one items contribute to a problem scale; higher scores indicate more social-emotional problems. The BITSEA has demonstrated acceptable to good internal and temporal consistency reliability, and there is evidence of convergent, discriminant, concurrent, and predictive validity (49, 50). In the present study, the BITSEA was administered during the 1- and 2- year post birth research visits, and the BITSEA total problem scale was used in the present study analyses (1-year post birth *α* = .77, 2-years post birth *α* = .82).

#### Maternal posttraumatic stress symptoms

Pre- and postnatal maternal PTSD symptoms were assessed via the 17-item, self-report, Posttraumatic Stress Disorder Checklist-Civilian Version [PCL-C; (51)]. Items are rated on a 5-point Likert-type scale ranging from 1 (*not at all)* to 5 (*extremely)* covering the experience of each symptom over the last month. Items are summed to create a total score; higher scores indicate greater symptom severity. The PCL-C has demonstrated high internal consistency as well as high 1-week and moderate 2-week test-retest reliability, and there is evidence for convergent validity (52, 53). The PCL-C was administered during the third trimester and 1-year post birth research visits, and the total score was used in the present study analyses (third trimester *α* = .87, 1-year post birth *α* = .91).

#### Maternal prenatal depression symptoms

Maternal prenatal depression symptoms were assessed via the 10-item, self-report, Edinburgh Postnatal Depression Scale [EPDS; (54)]. Items are rated on a 4-point scale ranging from 0 to 3 (score anchor descriptions differ based on the item) based on symptoms over the last 7 days. Items are summed to create a total score; higher scores indicate greater symptom severity. The EPDS has demonstrated test-retest reliability, internal consistency, construct validity, and convergent validity with other measures of depression (54, 55). In the present study, the EPDS was administered during the third trimester research visit, and the total score was used in the present study analyses (*α* = .76).

#### Maternal postnatal depression symptoms

Maternal postnatal depression symptoms were assessed via the 21-item, self-report, Beck Depression Inventory-II [BDI-II; (56)]. Items are rated on a 4-point scale ranging from 0 to 3 (score anchor descriptions differ based on the item) based on symptoms for the past 2 weeks. Items are summed to create a total score; higher scores indicate greater symptom severity. The BDI-II has demonstrated excellent internal consistency across a variety of samples along with high 1-week test-retest reliability, and acceptable construct validity (56, 57). In the present study, the BDI-II was administered during the 1-year post birth research visit, and the BDI-II total score was used in the present study analyses (*α* = .90).

#### Maternal anxiety symptoms

Pre- and postnatal maternal anxiety symptoms were assessed via the 6, self-report, anxiety items from the Brief Symptom Inventory [BSI; (58)]. Items are rated on a 5-point Likert-type scale ranging from 0 (*not at all*) to 4 (*extremel*y) based on how much symptoms have been bothersome or distressing during the past week. Items are summed to create a total score; higher scores indicate greater symptom severity. The BSI has demonstrated good internal consistency reliability, high 2-week test-retest reliability, and convergent validity (59, 60). In the present study, the BSI anxiety items were administered during the third trimester and 1-year post birth research visits, and the BSI anxiety total score was used in present study analyses (third trimester *α* = .77, 1-year post birth *α* = .81).

### Data analysis

Prior to mediation analyses, bivariate correlations between degree of disrupted prenatal maternal representations of the child, maternal mental health symptoms (PTSD, depression, and anxiety), and infant and toddler social-emotional functioning were examined. Mental health symptoms significantly associated with degree of disrupted prenatal maternal representations and infant and toddler social-emotional functioning were explored as mediators. Following past work examining degree of disrupted maternal representations and associations with mother and child variables (27), maternal age and income-to-needs ratio were explored as potential covariates, which were assessed via a demographics questionnaire at the third trimester (baseline) research visit. Of note, parity was also examined given that the sample included both primiparous (30%) and multiparous (70%) mothers. Mediation was tested in Mplus (version 7.4) where missing data were managed using MLR, a robust full-information maximum likelihood data estimation procedure. Standardized regression coefficients are presented for all paths in the model. Variables selected for model testing were evaluated for extreme outliers. For indirect effects, standardized coefficients and 95% confidence intervals are provided.

## Results

Prenatal PTSD symptoms were the only maternal mental health symptoms significantly associated with degree of disrupted prenatal maternal representations of the child and infant and toddler social-emotional functioning (see Table 1). Pre- and postnatal maternal depression symptoms and anxiety symptoms were each associated with both infant and toddler social-emotional functioning, but not with degree of disrupted prenatal maternal representations. Thus, only maternal prenatal PTSD symptoms were explored as a mediator in the relationship between degree of disrupted prenatal maternal representations and infant and toddler social-emotional functioning. No extreme outliers were present in the variables used in the final models.

**Table 1:** Bi-variate correlations between study variables.

Variable	1	2	3	4	5	6	7	8
1. Disrupted prenatal representations	1							
2. Infant social-emotional functioning	.20*	1						
3. Toddler social-emotional functioning	.28**	.73***	1					
4. Prenatal PTSD symptoms	.31**	.32**	.34**	1				
5. Postnatal PTSD symptoms	.13	.30**	.31**	.53***	1			
6. Prenatal depression symptoms	.11	.21*	.24*	.67***	.40***	1		
7. Postnatal depression symptoms	.10	.21*	.29**	.33**	.73***	.32**	1	
8. Prenatal anxiety symptoms	.06	.34***	.25*	.70***	.39***	.63***	.25**	1
9. Postnatal anxiety symptoms	.08	.28**	.23*	.41***	.76***	.46***	.68***	.41***

* *p* < .05. **p < .01. ***p < .001.

**Figure 1:**
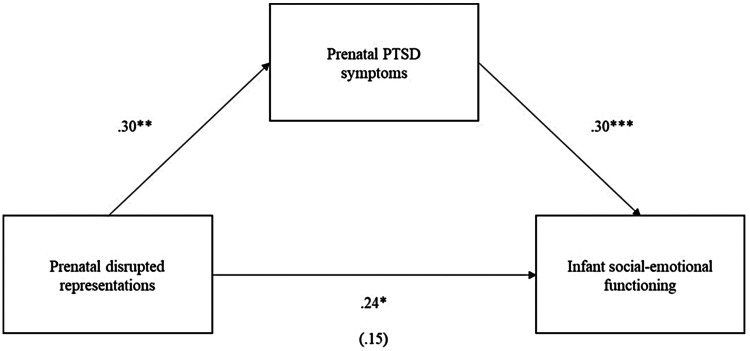
Mediation model 1 with prenatal PTSD symptoms significantly mediating the association between prenatal disrupted representations and infant social-emotional functioning (indirect effect = .09, se = .04, *p* = .012, 95% CI [0.02, 0.16]). All coefficients are standardized.

**Figure 2:**
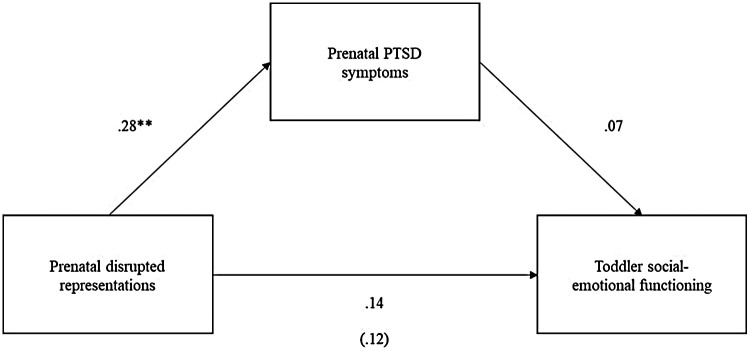
Mediation model 2 with no support for prenatal PTSD symptoms mediating the association between prenatal disrupted representations and toddler social-emotional functioning (indirect effect = .02, se = .02, *p* = .395, 95% CI [−0.02, 0.06]). All coefficients are standardized.

Income-to-needs ratio was inversely related to degree of disrupted prenatal maternal representations and included as a covariate in the mediation models. Maternal age and parity were not related to disrupted prenatal maternal representations and, thus, excluded from models. Two mediation models were tested. In the first model, infant social-emotional functioning was the target outcome to test more proximal associations between degree of disrupted prenatal maternal representations, maternal prenatal PTSD symptoms, and infant social-emotional functioning. Maternal prenatal PTSD symptoms mediated the association between degree of disrupted prenatal maternal representations and infant social-emotional functioning (see Figure 1). In the second model, toddler social-emotional functioning was the target outcome to test more distal associations, controlling for infant social-emotional functioning. Mediation was not supported; although degree of disrupted prenatal maternal representations predicted greater maternal prenatal PTSD symptoms, neither degree of disrupted prenatal maternal representations nor maternal prenatal PTSD symptoms significantly predicted toddler social-emotional functioning (see Figure 2).

**Figure 3:**
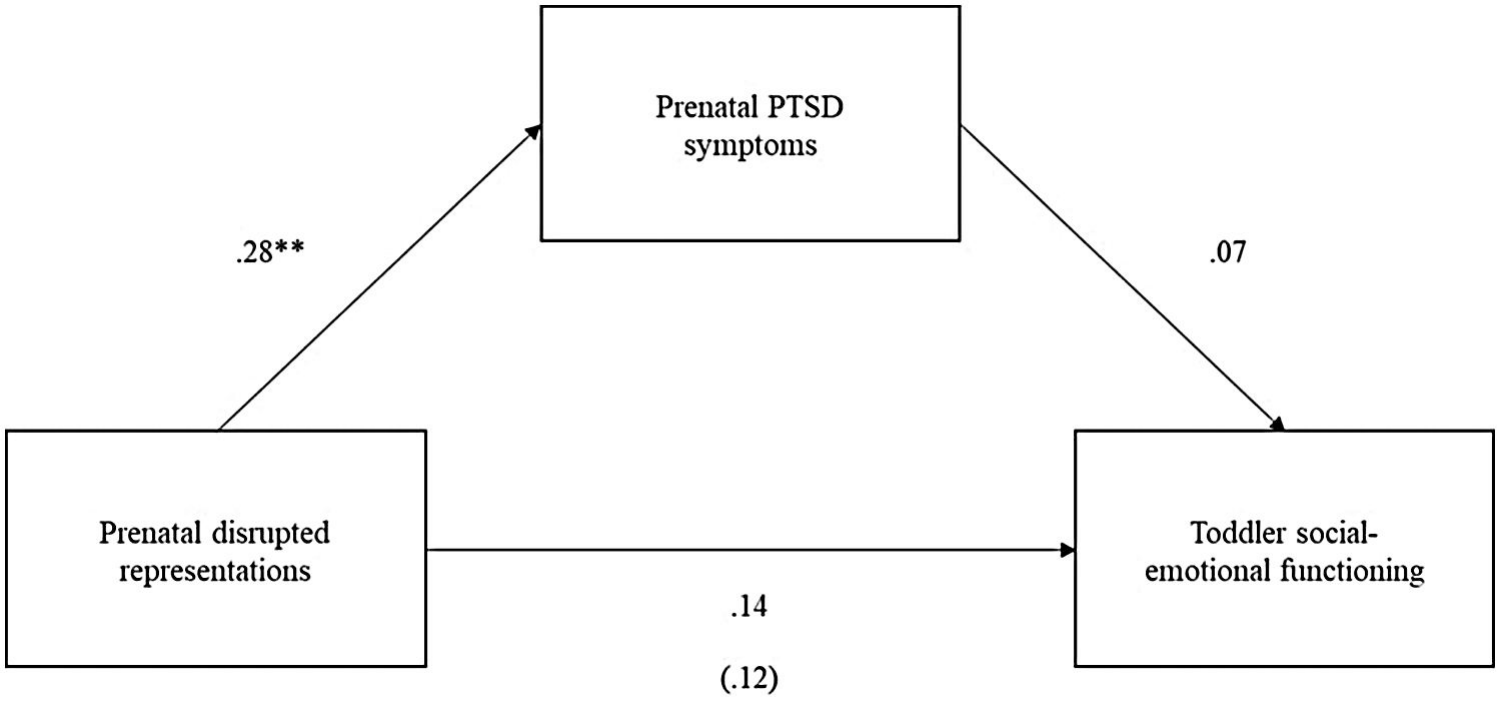
Exploratory mediation model 3 with no support for postnatal PTSD symptoms mediating the association between prenatal disrupted representations and toddler social-emotional functioning (indirect effect = .00, se = .01, *p* = .642, 95% CI [−0.01, 0.02]). All coefficients are standardized.

In a third exploratory model, maternal *postnatal* PTSD symptoms were explored as a mediator between degree of disrupted prenatal maternal representations and toddler social-emotional functioning to investigate whether maternal PTSD symptoms more proximal to the measure of social emotional-functioning may mediate the association. However, mediation was not supported and there were no significant paths (see Figure 3).

## Discussion

The present study sought to better understand the role of perinatal maternal mental health symptoms, particularly those that may be related to experiences of interpersonal trauma, such as PTSD, depression, and anxiety symptoms, in the association between degree of disrupted prenatal maternal representations of the child and infant and toddler social-emotional functioning. Based on previous research, it was hypothesized that perinatal (both third trimester and 1-year post birth) maternal PTSD, depression, and anxiety symptoms would each mediate the association between degree of disrupted prenatal maternal representations and infant and toddler social-emotional functioning

Interestingly, only prenatal maternal PTSD symptoms were significantly associated with degree of disrupted prenatal maternal representations of the child and infant and toddler social-emotional functioning, prompting an examination of PTSD symptoms as a mediator of the association between degree of disrupted prenatal maternal representations and infant and toddler social-emotional functioning. Upon further examination, only the association between degree of disrupted prenatal maternal representations and infant *but not toddler* social-emotional functioning was mediated by prenatal maternal PTSD symptoms. When maternal PTSD symptoms at 1-year post birth were explored as a possible mediator of the association between prenatal representations and toddler outcomes, mediation was not supported. These findings highlight both the significance of perinatal PTSD symptoms, in comparison to depression and anxiety symptoms, and the significance of PTSD symptoms during the *prenatal* period in elucidating the association between disrupted prenatal maternal representations and infant social-emotional functioning.

To the best of our knowledge, this is the first research study to examine maternal mental health symptoms during pregnancy and at 1-year post birth in relation to disrupted prenatal maternal representations of the child. Although previous research has found that distorted maternal representations are more likely when maternal depression, PTSD, or hostility symptoms are present (12, 33–37), our findings suggest that the same is not true for disrupted maternal representations, when measured continuously. Rather, it appears that there is something unique about PTSD symptoms during pregnancy in relation to degree of disrupted prenatal maternal representations of the child.

In the present study, we found an association between degree of disrupted prenatal maternal representations and maternal prenatal PTSD symptoms. In alignment with our previous research findings on the association between severity of mothers’ childhood interpersonal trauma and degree of disrupted prenatal maternal representations (19), it is possible this current finding reflects primarily unconscious cognitive, emotional, and behavioral impacts of unresolved childhood interpersonal trauma that may be further activated by pregnancy. The overlapping characteristics between disrupted maternal representations and childhood interpersonal trauma and PTSD symptoms are notable. For instance, childhood maltreatment generally involves contradictory, unpredictable experiences with the loved one/perpetrator. That is, experiences of care, comfort, protection, and love contrast with experiences of lack of care and safety, role or boundary confusion, withdrawal/distancing, and physical/verbal intrusiveness/abuse; these are similar characteristics to those that compose disrupted maternal representations. More subtle experiences of trauma [e.g., what (61) identified as “hidden trauma”] may be expressed, particularly to very young children, through unpredictable and unexplainable fearful and disoriented caregiver behavior. PTSD symptoms, similarly, broadly reflect intrusion (similar to, for example, intrusiveness and disorientation or dissociation in disrupted maternal representations), avoidance (similar to withdrawal), negative changes in cognitions and mood (similar to feelings of fear, fright, and helplessness, incoherency, and contradictory response styles in disrupted maternal representations), and significant changes in arousal and reactivity (similar to, for example, dysregulation). Further, pregnancy is highly activating and interpersonal in nature. It is a time when the mother-child relationship is developing, memories of relationships with primary caregivers are revisited, and current relationships are reorganized (62, 63). Intense psychological processes occur during pregnancy, such as transitioning from being a *receiver* of care to being a *provider* of care (64). Thus, the similarities between the underlying mental processes associated with disrupted maternal representations and interpersonal trauma and PTSD symptoms, along with the profound interpersonal and psychological experiences of pregnancy, may account for why PTSD symptoms, and not depression or anxiety symptoms, were associated with disrupted prenatal maternal representations. In short, pregnancy may heighten or activate unresolved childhood interpersonal trauma and related PTSD symptoms.

Previous research on organized maternal representations and mental health has focused on the postnatal time period (approximately 3 months to 20 months post birth). Like some other studies, we did not find any associations between disrupted *prenatal* maternal representations and *postnatal* maternal mental health symptoms, which may suggest that maternal representations, disrupted or otherwise, are more strongly associated with current mental health symptoms rather than past or future mental health symptoms. This also lends further support to the importance of assessing current maternal mental health symptoms as standard practice for interventions which aim to address maternal representations of the child and/or infant and early childhood social-emotional outcomes. Addressing maternal mental health symptoms may play a critical role in achieving treatment goals focused on maternal representations, the parent-child relationship, or infant and early childhood social-emotional outcomes. Indeed, previous research has found that mothers who report fewer prenatal depressive symptoms are more likely to experience shifts in maternal representations of the child from disengaged or distorted to balanced between pregnancy and 1-year post birth (65). Further research is needed to better understand the relation between timing of maternal mental health symptoms and maternal representations of the child.

Our findings also suggest that there is something unique about the development of disrupted maternal representations during the prenatal period and corresponding prenatal PTSD symptoms in relation to infant, rather than toddler, social-emotional functioning. The association between disrupted prenatal maternal representations and infant but not toddler social-emotional functioning was mediated by prenatal maternal PTSD symptoms. Further, neither prenatal nor postnatal maternal PTSD symptoms predicted toddler social-emotional functioning. These findings suggest that mothers’ prenatal psychological environment may have immediate implications for child social-emotional functioning in the first year of life. This not only supports the value of intervention during the prenatal period, as further discussed below, but also highlights the importance of screening for maternal PTSD symptoms during the prenatal period along with more common screenings for depression and anxiety symptoms. Screening for and addressing PTSD symptoms during the prenatal period may be a feasible, efficient, and effective intervention in the prevention of later difficulties in child social-emotional functioning. Further research in this area would be beneficial to better support interventions that provide parent-child services across the perinatal period.

For toddler social-emotional outcomes, it may be that the association with degree of disrupted prenatal maternal representations is mediated by caregiver variables that are more externalized, such as parenting behavior, rather than variables that are primarily internalized, such as PTSD symptoms. In fact, in our past work, we found that the association between degree of disrupted prenatal maternal representations and toddler social-emotional functioning was mediated by degree of disrupted caregiving behavior at 1-year post birth (27). Notably, degree of disrupted caregiving behavior did not mediate the association between degree of disrupted prenatal maternal representations and infant social-emotional functioning in this same sample. These results, in conjunction with the results from the present study, may suggest that while disrupted prenatal maternal representations hold implications for more proximal infant social-emotional functioning, it is the translation of representations into disrupted caregiver behavior that holds greater implications for toddler social-emotional functioning. It may also suggest that in the first year of life, when dependence on and proximity to the caregiver is highest, disrupted maternal representations impact infant social-emotional functioning primarily through the internalized maternal psychological environment. Then, when children become more mobile and autonomous in the second year of life, disrupted maternal representations may impact child social-emotional functioning primarily through caregiver behavior, particularly disrupted caregiver behavior which is conceptualized to be rooted in a history of interpersonal trauma (66).

Our findings support the importance of relationship-based, pre- and postnatal, parent-child interventions that address caregiver unresolved trauma and focus on caregiving representations as early as possible. Fraiberg (67) set in motion many such interventions and treatment models through the development of infant-parent psychotherapy, which incorporates treatment strategies that attend to the caregiver's internal world as it relates to the infant. For example, interventions may focus on resolving “ghosts in the nursery,” i.e., caregiver psychological conflicts that have led to an unconscious repetition of the past with one's child in the present, and making the unconscious conscious (68). Fraiberg's clinical work has formed the basis of or influenced several evidence-based, parent-child mental health home visiting and therapy models that can be implemented as early as pregnancy and have been shown to improve maternal mental health, maternal representations of the child, and infant and toddler development.

The Michigan model of Infant Mental Health-Home Visiting (IMH-HV) originated from Fraiberg's (67) infant-parent psychotherapy (69) and has demonstrated significant positive impacts for mothers and young children. Initial, open trial, research on the Michigan IMH-HV model demonstrated improvements in maternal sensitivity over time (70). Subsequently, based on a 12-month randomized controlled trial (RCT) with a sample of mothers (Medicaid-eligible with one or more parenting risk factors) who were either pregnant or who had a child between the ages of 0 and 24 months at baseline, research findings revealed that mothers who received IMH-HV services, in comparison to those who did not, reported a decrease in depression, anxiety, and PTSD symptoms over the course of services (71), had lower child abuse potential scores at 12 months (72), and evidenced improvements in parental reflective functioning (73). Further, RCT results indicated positive impacts on toddler development. Specifically, mothers who reported lower PTSD symptoms at the start of IMH-HV services, as compared to those who did not receive IMH-HV services and who reported subclinical PTSD symptoms at baseline, rated their toddlers’ social-emotional development more positively (74). Additionally, the Michigan IMH-HV model was found to buffer the negative impact of maternal history of childhood adversity on toddler language development (75).

Another intervention with its foundation in Fraiberg's (67) infant-parent psychotherapy is Child-Parent Psychotherapy [CPP; (76)], a widely disseminated, empirically supported, trauma treatment for young children and their caregivers. Notably, a perinatal adaptation of CPP demonstrated initial positive results among a sample of 64 pregnant women with a history of interpersonal trauma who completed perinatal CPP from the third trimester of pregnancy until their infants were 6 months old (77, 78). Specifically, results revealed a decrease in depression and posttraumatic stress symptoms and an increase in positive child-rearing attitudes from pre- to post-treatment.

Last, a RCT of psychoanalytic parent-infant psychotherapy with a sample of mothers experiencing mental health problems and social adversity and their infants (<12 months of age) demonstrated reductions in representational risk in the treatment group, but not the control group (79). Reductions were seen, specifically, in Helpless and Hostile subscale scores, both of which have some similarities to disrupted maternal representations of the child as assessed in this study. Lower levels of parenting stress over time, with notable reductions in perceived parent-child relational difficulties, as well as greater improvements in maternal depressive symptoms and overall sense of mastery, were also found for the treatment group in comparison to the control group in this RCT.

These empirical findings from several infant mental health home visiting and therapy models, along with the findings from this study, highlight the importance of relationship-based interventions that can begin prenatally and continue postnatally with mother-child dyads impacted by interpersonal trauma, mental health challenges, and/or adversity. Such interventions allow for treatment foci aimed at addressing internal psychological processes and problematic caregiving representations with potential for shifting attention to parenting at the behavioral level as needed, all while holding relationships at the center.

There are several strengths of the present study that are worth mentioning as well as a few limitations. The focus on a range of maternal mental health symptoms in relation to disrupted maternal representations of the child significantly expands existing knowledge about disrupted maternal representations. Furthermore, the longitudinal nature of the study allowed for better understanding of perinatal mental health symptoms across the prenatal and postnatal period and proximal (infant) and distal (toddler) social-emotional functioning in relation to other study variables. This allowed for the greater examination of how prenatal maternal representations might impact child social-emotional functioning across early development than has been done previously. Use of the WMCI to assess maternal representations of the child and the WMCI-D coding scheme to identify degree of disruption in prenatal maternal representations is also a strength. These tools offer a more objective and nuanced measure of the constructs assessed than self-report measures provide. For example, semi-structured interviews of maternal representations are likely less susceptible to social desirability, as it is harder to discern what the appropriate and socially expected response is. Further, semi-structured interviews allow for assessing not only the content of what one reports but also the process by which information is reported (80).

Regarding study limitations, utilizing mothers’ report of mental health symptoms and infant and early childhood social-emotional functioning may have introduced bias in the form of under- or over-reporting of information, as self- or caregiver- reports can be influenced by a range of factors (e.g., social desirability, limited self-awareness, or limited knowledge of normative or problematic child development; (81)). It would have been ideal to have also utilized structured clinical interviews to assess mental health symptoms and structured observational measures to assess infant and early childhood social-emotional functioning. Nonetheless, psychometrically strong self- and caregiver- reports were used as alternatives. Additionally, the study sample was composed of biological mothers; therefore, caution must be exercised in generalizing results and conclusions to other caregivers. Only one research team, to our knowledge, has published research on disrupted representations in other caregivers, specifically fathers (47, 82). Future research would benefit from conducting studies on disrupted representations of the child with not only fathers but also with other types of caregivers (e.g., adoptive parents, grandparents, foster parents).

Beyond those already mentioned, there are several additional models that would be beneficial for future research to explore. Continuing to explore factors that mediate the impact of disrupted maternal representations of the child on infant and early childhood social-emotional functioning would not only provide greater understanding of these constructs but would also inform interventions by providing reliable targets where intervention may be more beneficial. Other important factors to explore include current interpersonal history of trauma and/or loss, (including trauma and loss specific to childbirth), sociocultural stressors, social isolation, and emotion regulation, to name a few. Exploring moderators of the association between degree of disrupted maternal representations and infant and early childhood social-emotional functioning would also be beneficial. Although research has found that disrupted maternal representations of the child are highly stable and resistant to short-term intervention (i.e., participation in an 8-week attachment-focused parenting group; (21)), it would still be beneficial to explore whether service utilization, in general, plays a moderating role. Identifying which services or interventions are most impactful in shifting the association between disrupted maternal representations and infant and toddler social-emotional functioning would provide critical information. Given the important role of social support during the perinatal period in relation to maternal mental health symptoms (83–85) and infant and toddler social-emotional functioning (86, 87), exploring social support or protective relational contexts as moderators of mental health mediators may provide information that further guides interventions. Last, continuing to expand the knowledge base on disrupted maternal representations of the child, including risk and protective factors, stability and change, and outcomes for this highly vulnerable group of caregivers, is important to understanding this construct and to engaging in assessment and interventions for disrupted maternal representations in the most effective and efficient manner.

In sum, our findings highlight the significance of maternal PTSD symptoms during the prenatal period in the relationship between disrupted prenatal maternal representations of the child and infant social-emotional functioning. The findings further demonstrate the impact of maternal trauma on infant development, which is a complex process. Our findings suggest that maternal history of interpersonal trauma can lead to increased risk for PTSD symptoms, and, importantly, disrupted representations of the child during pregnancy, which, in turn, can impact child social-emotional functioning during infancy. Thus, it is important that screening and treatment of maternal PTSD symptoms be included as part of standard practice of care when providing clinical interventions to perinatal mothers and very young children, ideally beginning during pregnancy. Further, our findings highlight the value of research during the prenatal period and assessing child development as it unfolds over time alongside various internal and external caregiver factors. This research plays an important role in developing and promoting interventions that benefit mothers, young children, and the mother-child relationship as early as possible.

## Data Availability

The raw data supporting the conclusions of this article will be made available by the authors, without undue reservation.
